# Implant Optimisation for Primary Hip Replacement in Patients over 60 Years with Osteoarthritis: A Cohort Study of Clinical Outcomes and Implant Costs Using Data from England and Wales

**DOI:** 10.1371/journal.pone.0140309

**Published:** 2015-11-12

**Authors:** Simon S. Jameson, James Mason, Paul N. Baker, Paul J. Gregg, David J. Deehan, Mike R. Reed

**Affiliations:** 1 School of Medicine, Pharmacy and Health, Durham University, Queen's Campus, University Boulevard, Stockton-on-Tees, TS17 6BH, United Kingdom; 2 The National Joint Registry for England and Wales, London, United Kingdom; 3 Department of Orthopaedic Surgery, South Tees Hospitals NHS Foundation Trust, Marton Road, Middlesbrough, TS4 3BW, United Kingdom; 4 Department of Orthopaedic Surgery, Newcastle Hospitals NHS Foundation Trust, Freeman Road, High Heaton, Newcastle upon Tyne, NE7 7DN, United Kingdom; 5 Department of Orthopaedic Surgery, Northumbria Healthcare NHS Foundation Trust, Woodhorn Lane, Ashington, Northumberland, NE63 9JJ, United Kingdom; University of Zaragoza, SPAIN

## Abstract

**Background:**

Hip replacement is one of the most commonly performed surgical procedures worldwide; hundreds of implant configurations provide options for femoral head size, joint surface material and fixation method with dramatically varying costs. Robust comparative evidence to inform the choice of implant is needed. This retrospective cohort study uses linked national databases from England and Wales to determine the optimal type of replacement for patients over 60 years undergoing hip replacement for osteoarthritis.

**Methods and Findings:**

Implants included were the commonest brand from each of the four types of replacement (cemented, cementless, hybrid and resurfacing); the reference prosthesis was the cemented hip procedure. Patient reported outcome scores (PROMs), costs and risk of repeat (revision) surgery were examined. Multivariable analyses included analysis of covariance to assess improvement in PROMs (Oxford hip score, OHS, and EQ5D index) (9159 linked episodes) and competing risks modelling of implant survival (79,775 procedures). Cost of implants and ancillary equipment were obtained from National Health Service procurement data.

**Results:**

EQ5D score improvements (at 6 months) were similar for all hip replacement types. In females, revision risk was significantly higher in cementless hip prostheses (hazard ratio, HR = 2.22, p<0.001), when compared to the reference hip. Although improvement in OHS was statistically higher (22.1 versus 20.5, p<0.001) for cementless implants, this small difference is unlikely to be clinically important. In males, revision risk was significantly higher in cementless (HR = 1.95, p = 0.003) and resurfacing implants, HR = 3.46, p<0.001), with no differences in OHS. Material costs were lowest with the reference implant (cemented, range £1103 to £1524) and highest with cementless implants (£1928 to £4285).

Limitations include the design of the study, which is intrinsically vulnerable to omitted variables, a paucity of long-term implant survival data (reflecting the duration of data collection), the possibility of revision under-reporting, response bias within PROMs data, and issues associated with current outcome scoring systems, which may not accurately reflect level of improvement in some patients.

**Conclusions:**

Cement fixation, using a polyethylene cup and a standard sized head offers good outcomes, with the lowest risks and at the lowest costs. The most commonly used cementless and resurfacing implants were associated with higher risk of revision and were more costly, while perceptions of improved function and longevity were unsupported.

## Introduction

Management of osteoarthritis (OA) of the hip is a significant global health burden. Hip replacement is an established and successful treatment of end-stage OA, with excellent quality of life improvement and cost-effectiveness [1,2]. Over 270,000 hip replacements are performed in the United States (US) annually, and almost 90,000 within the United Kingdom (UK) [3,4,5]. The national tariff for a hip replacement is £5280 in England. This equates to approximately £475million in annual UK healthcare costs. These costs are expected to triple over the next five years, whilst annual volume is expected to double within ten [6].

Cemented hip replacements (which utilise a polymer known as ‘cement’ to secure the implant in place) with a metal-on-polyethylene (MoP) articulating (‘bearing’) surface account for one third of all hip replacements implanted in England and Wales since 2003. These devices show consistently good implant survival in long-term cohort studies and worldwide joint replacement registries [3,6,7,8,9,10,11,12,13,14,15,16,17,18]. They utilise tried and tested technology, and are inexpensive. However, concerns of early loosening and implant failure during the 1980s [19,20,21,22,23] drove the development of cementless implants, which rely on press-fit stability and bone integration for fixation rather than cement [24]. Advances in engineering also led to a proliferation of implant options available within brands; larger, more anatomical femoral head sizes in an attempt to reduce dislocation risk, and ‘hard’ articulations, where highly engineered metal-on-metal (MoM) or ceramic-on-ceramic (CoC) bearings are employed in an effort to minimise long-term wear and subsequent failure [25,26,27]. Cementless implants now account for the majority of replacements in North America and Australia, and their use in England and Wales has recently surpassed cemented implants [3,28,29]. Resurfacing devices, which resurface the femoral head and preserve bone (rather than excising femoral head/neck and replacing with a ball and stem, as in standard hip replacement), provide near anatomically-sized components and were introduced in the 1990s with the aim of reducing dislocation risk, improving function and allowing an ‘easier’ revision if required [30]. These were designed predominantly for younger patients, but surgeons widened their indications as good early results encouraged use in older patients. Although there is little data on implant costs in the literature, there is a logical perception that implants with modular components (providing numerous options), modern technologies and complex, highly engineered components are more costly. Despite this, thorough evaluation of the evidence for different types of hip replacement is absent from the literature.

Some patients with hip replacements will require a revision procedure to replace a failed or worn implant. The National Joint Registry (NJR) was established in 2003 to provide a record of hip replacements and any subsequent revisions performed in the pubic and private health systems in England and Wales. Patient Reported Outcomes Measures (PROMs) have been collected on hip replacement patients in the public system since 2008. Linkage of these national datasets allows the analysis of patient functional outcome following hip replacement and subsequent implant failure rates for specific implants. Taking the most commonly used cemented hip replacement as the reference implant for comparison, the objective of this study was to provide a summative evaluation of different implant types in order to determine the most cost-effective components for hip replacement, referencing patient reported outcomes and risk of implant revision. This study examines the eighty percent of all primary hip replacements that are performed in patients 60 years and over [3]. Younger patients (under 60 years is arbitrarily a reasonable threshold) may have differing demands of their prostheses, and as such have been analysed elsewhere [31].

## Methods

### Design

A retrospective cohort study design assessed prospectively collected patient-level PROMs and NJR data to compare outcomes and implant survival across different primary hip replacements, with supplementary material costs for specific implant combinations obtained through National Health Service (NHS) procurement.

### Data

The single most commonly used brands of each type of hip replacement performed in England and Wales were chosen for the analysis, in order to control for brand heterogeneity within each type (the NJR annual report provides adequate analysis of the entire breadth of replacements available–our intention was to specifically analyse component options within brands, which would be impossible across all brands). Individual analyses of the same data on each individual hip replacement type have already defined component options within brand that confer the lowest revision risk (i.e. the longest survival) [32,33,34,35]. For this current analysis we stratified each hip replacement type based on these previously established component revision risks into ‘optimal’ component sets (with significantly lower revision risk) and ‘sub-optimal’ (all remaining component options) (**Table 1**).

**Table 1:** Implants studied by type of hip replacement, with descriptions of optimal and sub-optimal component configurations.

Type	Brand combination	Manufacturer	Market share, by type (England & Wales)
Cemented	Exeter V40 stem	Stryker Orthopaedics, Mahwah, New Jersey, United States	23%
	Contemporary polyethylene cup		
	*Optimal component set*:		
	Any Exeter stem		
	Flanged version of Contemporary cup		
	28mm or 32mm femoral head (metal or ceramic*)		
	*Sub-optimal component set*:		
	Small heads (<28mm)		
	Hooded version of Contemporary cup		
Cementless	Corail stem	DePuy Ltd, Leeds, United Kingdom	31%
	Pinnacle modular (shell and liner) cup		
	*Optimal component set*:		
	Medium/large Corail stem (size 11 or greater)		
	Pinnacle cup / polyethylene liner (metal or ceramic head*)		
	*Sub-optimal component set*:		
	Small Corail stems (<size 11)		
	Pinnacle metal and ceramic liners*		
Hybrid	Exeter V40 stem	Stryker Orthopaedics, Mahwah, New Jersey, United States	33%
	Trident modular (shell and liner) cup		
	*Optimal component set*:		
	Any Exeter stem		
	Solid shell Trident cup		
	Ceramic bearing or a XLPE liner (metal or ceramic head*)		
	*Sub-optimal component set*:		
	Cluster hole Trident shell		
	Conventional polyethylene liner		
Resurfacing	Birmingham Hip Resurfacing (BHR)	Smith & Nephew, Memphis, Tennessee, United States	55%
	*Optimal component set*:		
	Components with head size of 48mm or greater		
	*Sub-optimal component set*:		
	Components with head size <48mm		

*grouped together as no significant benefit of options was identified, XLPE–highly cross-linked polyethylene

All primary hip replacements performed using the specified implants on patients over 60 years and submitted to the NJR between 1^st^ April 2003 and 31^st^ December 2010 were initially included. Subsequently, exclusion criteria were employed as follows: all procedures with an indication other than OA; procedures with missing implant or patient data; and rarely used implant options [32,33,34,35].

**Fig 1:**
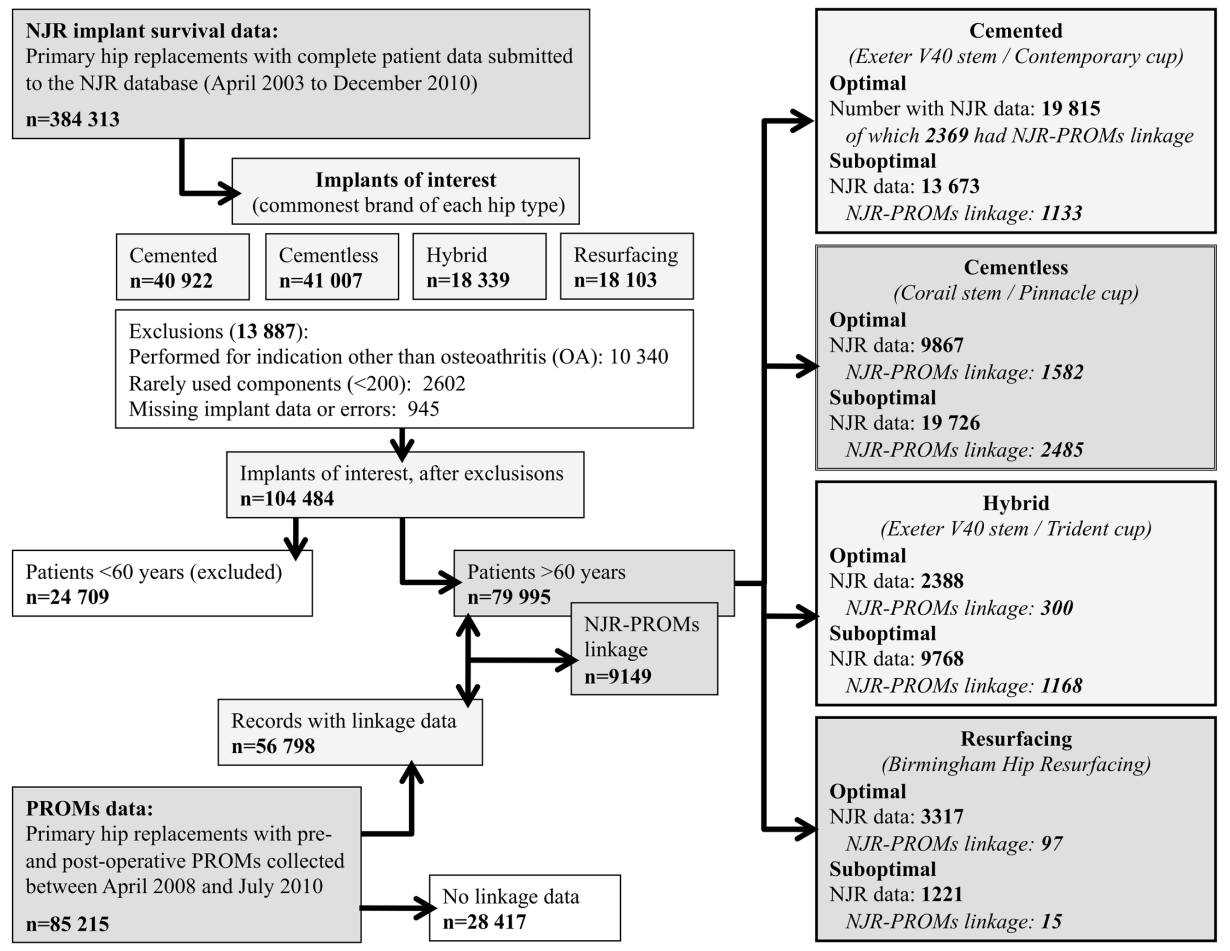
Flowchart describing inclusion criteria and study populations.

The national PROMs project uses validated measures of hip-specific (Oxford hip score [OHS]) [36] and general health status outcomes (EuroQol [EQ-5D-3L]) [37] collected pre- and around six months post-operatively. By linking databases at the patient level, PROMs data can be combined with the corresponding demographic and operative details held in the NJR. The study population is summarised in **Fig 1**. The demographic, surgical and implant-related variables available for analysis are listed in **S1 Table**.

For this analysis PROMs of interest were improvements between the pre- and post-operative scores (the ‘change scores’) and self-reported readmission and reoperation in the post-operative period. Change scores, being approximately normally distributed, are analytically preferable to post-operative scores [38]. The OHS (scored 0 lowest to 48 highest) has previously been shown to be a reliable, valid and responsive outcome measure for patients with hip OA undergoing replacement surgery [39]. The EQ-5D index (scored 0 to 1, where 0 is no health [i.e. dead] and 1 is perfect health) is a measure of health status used for clinical and economic appraisal. It evaluates five different aspects of general health (mobility, self-care, usual activities, pain/ discomfort and anxiety/depression) that are scored and combined using population weightings to produce a single index value for health status [37]. In this context, readmission and reoperation are used as a crude surrogate marker for hip dislocation. Dislocation occurs when the femoral component disarticulates from within the acetabular component. This is an acute event that requires readmission and manipulation under anaesthesia to restore normal component positions. Unfortunately this data is not captured by the NJR, but may vary depending on head size and bearing material. Thus, to provide a summative evaluation, it is reasonable to include these measures, despite the limitations. Within the pre-operative PROMs questionnaire, patients are also asked about comorbidities, general health and self-reported disability. These can be used to adjust for differences in health status between patient groups.

### Statistical Analysis

Implants were compared based on previously stratified revision risk within prosthesis types. Therefore, eight groups were compared (four ‘optimal’ groups and four ‘sub-optimal’ groups) (**Fig 1**). Differences in baseline characteristics across the groups were analysed using one-way analysis of variance test (ANOVA, parametric continuous data variables), the Kruskal-Wallis test (non-parametric continuous data variables) or the Chi-square test (categorical data variables).

Univariable analysis was performed initially to identify variables potentially influencing each outcome, based on statistical rejection criteria of p>0.10; these variables were then included in the multivariable models (see supplementary material for complete statistical methods). Due to the large population sizes and the questionable merits of statistically adjusting for gender, we chose to analyse data on males and females separately.

Implant survival times for patients who had not undergone revision were censored on the 31^st^ December 2010. Competing risks models were used to adjust for potential differences in mortality across the implant groups, where patient death prior to either revision or censoring was the competing risk [40]. Cumulative incidence charts were then produced for each type of implant and by gender. Analysis of covariance (ANCOVA) was used for testing differences in OHS and EQ5D index change scores. Multivariable logistic regression was used to analyse differences in the risk of readmission and reoperation. Time from implantation to questionnaire completion was included in models to evaluate whether differences in duration of follow-up influenced findings. Pre-operative scores were included within all models, as recommended by the designers of the OHS [39].

Results of the survival analysis were presented as hazard ratios (HRs). Statistical models for the change scores were evaluated with the margins function in STATA in order to provide predicted values separately for each of the implant groups. P-values are provided as statistical tests of the differences between the reference implant and the seven others. Significance was taken as p<0.05. All values are provided with 95% confidence intervals (CIs): ratios greater than one indicate that risk is higher when compared with the reference category. All models were fitted using STATA 12 (StataCorp LP, Texas, USA). Further supplementary information is available in **S1 Text** and **S2 to S5 Tables**.

Costs for specific implant combinations were provided by NHS Wales (all seven hospital Trusts) and NHS supply chain (buyers on behalf of 30 hospital Trusts within the English NHS). Highest and lowest prices paid for implants during 2012 are provided for each of the implant components. A mode cost was also produced at source and provided. These costs represent actual prices paid, after discounts. In addition, the NJR levy fee (£20, which is included in the amount paid for each implant) and Value Added Tax (VAT, at 20%) were added for the total costs. The costs presented in this study also include acetabular screws (for cementless cup fixation) when used, the commonest cement used for each implant type, femoral cement restrictors and all equipment required to mix and perform pressurised cementation. Although it is acknowledged that hip replacement with cementless implants may result in slightly shorter operative time, for the purposes of this analysis it is assumed that theatre utilisation and length of stay was similar for all types of replacement, and that differences in specific implant costs approximated to incremental costs.

### Ethics

The National Joint Registry (England and Wales) Research Committee approved this study. Explicit patient consent is taken at the time of data collection for both the NJR and PROMs. Further ethical approval was not required for this study. Patient records/information was anonymized and de-identified prior to receipt of data and analysis.

## Results

There were 79,775 procedures available for implant survival analysis within the NJR dataset. Significant baseline differences were seen in age, ASA grade, proportions of females and BMI for the type of implant received (**Table 2**). Linkage of PROMs data with data stored in the NJR dataset was possible in 9159 procedures. The demographics of patients and implants for the linked procedures were qualitatively similar to the NJR population (**Table 3**). Unadjusted pre-operative OHS and EQ5D index scores were clinically similar across the cemented, cementless and hybrid replacements, but higher prior to resurfacings (**Table 4**). Post-operative scores were lowest in the sub-optimal cemented group and highest after any resurfacing.

**Table 2:** Patient demographics for National Joint Registry population studied, by implant group.

	Cemented		Hybrid		Cementless		Resurfacing		Difference
	Optimal	Sub-opt.	Optimal	Sub-opt.	Optimal	Sub-opt.	Optimal	Sub-opt.	
Number (%)	19815 (24.8)	13673 (17.1)	2388 (3.0)	9768 (12.2)	9867 (12.4)	19726 (24.7)	3317 (4.2)	1221 (1.5)	
Age, median years (range)	74.8 (60 to 100)	74.8 (60 to 97)	67.6 (60 to 97)	71.7 (60 to 103)	72.2 (60 to 98)	68.7 (60 to 106)	63.8 (60 to 89)	63.3 (60 to 88)	p<0.001
Female	12788 (64.5)	9163 (67.0)	1238 (51.8)	6142 (62.9)	5303 (53.7)	11559 (58.6)	166 (5.0)	872 (71.4)	p<0.001
ASA									
1	2461 (12.4)	1822 (13.3)	508 (21.3)	1336 (13.7)	1219 (12.4)	2921 (14.8)	1343 (40.5)	542 (44.4)	p<0.001
2	13835 (69.8)	9496 (69.5)	1637 (68.6)	6888 (70.5)	7186 (72.8)	14280 (72.4)	1833 (55.3)	644 (52.7)	
3+	3519 (17.8)	2355 (17.2)	243 (10.2)	1544 (15.8)	1462 (14.8)	2525 (12.8)	141 (4.3)	35 (2.9)	
BMI, mean kg/m^2^(sd, range)	28.3 (5.0, 15 to 63)	27.9 (5.0, 15 to 65)	28.4 (5.1 16 to 56)	28.1 (5.1, 15 to 61)	28.4 (5.1, 15 to 64)	28.5 (5.2, 15 to 64)	27.8 (4.3, 18 to 64)	27.3 (4.2, 18 to 40)	p = 0.015

ASA–American Society of Anesthesiologists, BMI–body mass index (data based on 34756 procedures [44%])

Statistical notes: one-way analysis of variance (ANOVA) used for parametric data, Kruskal-Wallis test for non parametric data, Chi squared test for proportions

**Table 3:** Patient demographics for National Joint Registry-PROMs linked population studied, by implant group.

	Cemented		Hybrid		Cementless		Resurfacing		Difference
	Optimal	Sub-opt.	Optimal	Sub-opt.	Optimal	Sub-opt.	Optimal	Sub-opt.	
Number (%)	2369 (25.9)	1133 (12.4)	300 (3.3)	1168 (12.8)	1582 (17.3)	2485 (27.2)	97 (1.1)	15 (0.2)	
Age, median years (range)	74.0 (60 to 93)	75.2 (60 to 94)	68.1 (60 to 91)	71.6 (60 to 93)	72.0 (60 to 95)	67.8 (60 to 96)	64.2 (60 to 75)	62.8 (60 to 67)	p<0.001
Female	1463 (61.8)	747 (65.9)	164 (54.7)	744 (63.7)	776 (49.1)	1425 (57.3)	1 (1.0)	13 (86.7)	p<0.001
ASA									
1	213 (9.0)	96 (8.5)	53 (17.7)	122 (10.5)	162 (10.2)	345 (13.9)	35 (36.1)	5 (33.3)	p<0.001
2	1709 (72.1)	829 (73.2)	217 (72.3)	888 (76.0)	1201 (75.9)	1897 (76.3)	59 (60.8)	10 (66.6)	
3+	447 (18.9)	208 (18.4)	30 (10.0)	158 (13.5)	219 (13.8)	243 (9.8)	3 (3.1)	0 (0)	
BMI, mean kg/m (sd, range)	28.6 (5.0, 16 to 55)	28.1 (4.7, 15 to 46)	28.4 (4.6 17 to 44)	28.2 (4.8, 17 to 43)	28.5 (4.9, 16 to 56)	28.6 (5.2, 15 to 50)	28.0 (4.0, 20 to 38)	27.8 (2.9, 23 to 32)	p = 0.679

PROMs–patient reported outcome measures, ASA–American Society of Anesthesiologists, BMI–body mass index (data based on 5843 procedures [64%])

Statistical notes: one-way analysis of variance (ANOVA) used for parametric data, Kruskal-Wallis test for non parametric data, Chi squared test for proportions

**Table 4:** Patient reported outcomes for populations studied, by implant group and gender.

	Cemented		Hybrid		Cementless		Resurfacing		p value
	Optimal	Sub-optimal	Optimal	Sub-optimal	Optimal	Sub-optimal	Optimal	Sub-optimal	
Females (n, %)	1463 (27.4)	747 (14.0)	164 (3.1)	744 (14.0)	776 (14.6)	1425 (26.7)	1 (0.0)	13 (0.2)	
**Oxford Hip scores**									
Pre-operative, mean (sd,range)	17.4 (8.0, 0 to 44)	16.8 (8.0, 0 to 42)	19.7 (7.8, 4 to 37)	18.3 (8.0, 1 to 38)	17.3 (7.7, 1 to 43)	18.5 (8.1, 0 to 44)	13	25.9 (4.5,18 to 33)	<0.001
Post-operative, median (range)	40 (4 to 48)	38 (2 to 48)	43 (13 to 48)	42 (5 to 48)	42 (6 to 48)	42 (2 to 48)	48	46 (21 to 48)	<0.001
**EQ5D index**									
Pre-operative, mean (sd, range)	0.342 (0.313, -0.43 to 1)	0.319 (0.325, -0.48 to 1)	0.432 (0.301, -0.24 to 0.88)	0.356 (0.323, -0.59 to 1)	0.346 (0.317, -0.35 to 1)	0.366 (0.318, -0.59 to 1)	0.516	0.586 (0.192, 0.09 to 0.76)	0.008
Post-operative, median (range)	0.796 (-0.24 to 1)	0.760 (-0.24 to 1)	0.850 (-0.18 to 1)	0.814 (-0.24 to 1)	0.812 (-0.13 to 1)	0.796 (-0.32 to 1)	1	1 (0.52 to 1)	<0.001
**Time from op to PROMs complete**, mean days (sd, range)	208.8 (29.0, 183 to 358)	209.5 (29.2, 183 to 358)	209.5 (30.6, 184 to 360)	209.4 (28.5, 183 to 364)	207.2 (25.8, 185 to 357)	208.3 (28.4, 183 to 360)	193	258.8 (46.8, 192 to 316)	0.323
Males (n, %)	906 (23.7)	386 (10.1)	136 (3.6)	424 (11.1)	806 (21.1)	1060 (27.8)	96 (2.5)	2 (0.1)	
**Oxford Hip scores**									
Pre-operative, mean (sd,range)	19.8 (7.9, 0 to 44)	19.1 (8.1, 2 to 48)	22.1 (7.9, 4 to 41)	20.4 (8.5, 2 to 42)	19.9 (8.0, 2 to 42)	20.4 (8.3, 3 to 44)	25.7 (8.2, 4 to 43)	21.5 (0.7,21 to 22)	0.001
Post-operative, median (range)	43 (7 to 48)	41 (12 to 48)	44 (14 to 48)	43 (11 to 48)	43 (2 to 48)	44 (1 to 48)	45 (13 to 48)	48	<0.001
**EQ5D index**									
Pre-operative, mean (sd, range)	0.425 (0.300, -0.32 to 1)	0.439 (0.288, -0.48 to 0.88)	0.439 (0.288, -0.07 to 0.80)	0.422 (0.302, -0.35 to 1)	0.418 (0.301, -0.35 to 1)	0.425 (0.311, -0.35 to 1)	0.551 (0.253, -0.18 to 81)	0.516	0.016
Post-operative, median (range)	0.814 (-0.18 to 1)	0.814 (-0.18 to 1)	0.883 (0.88 to 1)	1 (-0.24 to 1)	0.850 (-0.18 to 1)	0.883 (-0.59 to 1)	1 (-0.02 to 1)	1	<0.001
**Time from op to PROMs complete**, mean days (sd, range)	208.2 (28.5, 183 to 363)	207.6 (27.2, 183 to 355)	208.5 (27.1, 183 to 336)	205.0 (22.3, 184 to 355)	207.9 (28.7, 183 to 363)	207.2 (27.5, 183 to 362)	272.4 (44.3, 184 to 336)	195.5 (3.5, 193 to 198)	0.192

SD–standard deviation, PROMs–patient reported outcome measures

Statistical notes: one-way analysis of variance (ANOVA) used for parametric data, Kruskal-Wallis test for non parametric data, Chi squared test for proportions

### Patient Reported Outcome Measures

In females OHS change was significantly higher (22.1 versus 20.5, p<0.001) in the optimal cementless group when compared with the reference implant. No other implant combination had a significantly better OHS improvement. There were no significant OHS improvement benefits across the implant types in males. No implant combination displayed an EQ5D index improvement significantly greater than the reference, in either sex (**Table 5**). For OHS, 40% to 42% of variation within the models could be explained by known variables; for EQ5D index this was 61% to 63% (S4 Table). There were no significant differences in readmission or further surgery (**Table 6**).

**Table 5:** Patient reported outcome scores following hip replacement in patients aged 60 years and over (simple and multivariable analyses).

	Simple			Multivariable		
	Value	95% CI	P value	Value	95% CI	P value
**Females (n = 5333)**						
**Change in OHS**						
Optimal cemented (n = 1463)	20.2	19.7 to 20.7	Reference	20.5	20.1 to 21.0	Reference
Sub-optimal cemented (n = 747)	19.2	18.4 to 19.9	0.029	19.7	19.0 to 20.5	0.075
Optimal hybrid (n = 164)	20.4	18.9 to 21.9	0.773	21.7	20.0 to 23.4	0.207
Sub-optimal hybrid (n = 744)	20.7	20.0 to 21.4	0.227	20.9	20.1 to 21.6	0.463
Optimal cementless (n = 776)	21.9	21.2 to 22.6	<0.001	22.1	21.3 to 22.8	<0.001
Sub-optimal cementless (n = 1425)	20.7	20.2 to 21.2	0.169	21.0	20.4 to 21.5	0.270
**Change in EQ5D index**						
Optimal cemented (n = 1463)	0.421	0.402 to 0.439	Reference	0.426	0.414 to 0.439	Reference
Sub-optimal cemented (n = 747)	0.429	0.403 to 0.454	0.619	0.418	0.398 to 0.439	0.502
Optimal hybrid (n = 164)	0.373	0.320 to 0.427	0.103	0.452	0.404 to 0.499	0.312
Sub-optimal hybrid (n = 744)	0.433	0.408 to 0.459	0.421	0.436	0.416 to 0.457	0.430
Optimal cementless (n = 776)	0.446	0.421 to 0.471	0.100	0.447	0.427 to 0.467	0.086
Sub-optimal cementless (n = 1425)	0.417	0.398 to 0.435	0.765	0.420	0.404 to 0.435	0.182
**Males (n = 3826)**						
**Change in OHS**						
Optimal cemented (n = 906)	20.1	19.5 to 20.7	Reference	20.3	19.7 to 20.9	Reference
Sub-optimal cemented (n = 386)	20.4	19.5 to 21.4	0.553	19.9	18.9 to 20.9	0.521
Optimal hybrid (n = 136)	20.0	18.3 to 21.6	0.882	18.9	17.2 to 20.6	0.140
Sub-optimal hybrid (n = 424)	20.5	19.6 to 21.4	0.488	20.6	19.7 to 21.5	0.603
Optimal cementless (n = 806)	20.7	20.0 to 21.3	0.222	20.6	19.9 to 21.3	0.521
Sub-optimal cementless (n = 1060)	20.2	19.6 to 20.8	0.820	19.8	19.1 to 20.5	0.295
Optimal resurfacing (n = 96)	17.1	15.2 to 19.0	0.004	19.1	17.2 to 21.1	0.282
**Change in EQ5D index**						
Optimal cemented (n = 906)	0.379	0.357 to 0.401	Reference	0.390	0.374 to 0.407	Reference
Sub-optimal cemented (n = 386)	0.417	0.384 to 0.450	0.060	0.391	0.364 to 0.418	0.988
Optimal hybrid (n = 136)	0.377	0.322 to 0.432	0.941	0.364	0.316 to 0.411	0.302
Sub-optimal hybrid (n = 424)	0.419	0.387 to 0.450	0.044	0.415	0.389 to 0.441	0.121
Optimal cementless (n = 806)	0.395	0.371 to 0.418	0.345	0.401	0.381 to 0.421	0.428
Sub-optimal cementless (n = 1060)	0.390	0.370 to 0.410	0.482	0.358	0.340 to 0.377	0.011
Optimal resurfacing (n = 96)	0.340	0.273 to 0.406	0.270	0.398	0.343 to 0.453	0.790

OHS–Oxford Hip Score, CI–confidence interval

Note: No predicted values are available for resurfacings in females (14 PROMs available only) and others resurfacing in males (2 PROMs only)

**Table 6:** Risk of readmission and reoperation following hip replacement in patients aged 60 years and over (simple and multivariable analyses).

		Simple			Multivariable		
	Number (%)	OR	95% CI	P value	OR	95% CI	P value
**Females (n = 5333)**							
**Readmission**							
Optimal cemented (n = 1463)	92 (6.3)	1			1		
Sub-opt. cemented (n = 747)	67 (8.9)	1.47	1.06 to 2.04	0.022	1.67	1.11 to 2.51	0.013
Optimal hybrid (n = 164)	8 (4.9)	0.76	0.36 to 1.60	0.477	1.76	0.23 to 2.50	0.651
Sub-optimal hybrid (n = 744)	47 (6.3)	1.00	0.70 to 1.44	0.979	1.24	0.77 to 2.00	0.379
Optimal cementless (n = 776)	56 (7.2)	1.16	0.82 to 1.64	0.401	1.25	0.79 to 1.98	0.340
Sub-opt cementless (n = 1425)	82 (5.8)	0.91	0.67 to 1.24	0.547	1.18	0.78 to 1.78	0.423
**Reoperation**							
Optimal cemented (n = 1463)	29 (2.0)	1			1		
Sub-opt. cemented (n = 747)	20 (2.7)	1.36	0.76 to 2.42	0.296	1.22	0.67 to 2.22	0.522
Optimal hybrid (n = 164)	3 (1.8)	0.92	0.28 to 3.06	0.894	0.99	0.29 to 3.31	0.982
Sub-optimal hybrid (n = 744)	15 (2.0)	1.02	0.54 to 1.91	0.957	0.95	0.50 to 1.82	0.879
Optimal cementless (n = 776)	6 (0.8)	0.39	0.16 to 0.93	0.034	0.46	0.16 to 1.35	0.156
Sub-opt cementless (n = 1425)	27 (1.9)	0.96	0.56 to 1.62	0.865	0.83	0.47 to 1.46	0.519
**Males (n = 3826)**							
**Readmission**							
Optimal cemented (n = 906)	88 (9.7)	1			1		
Sub-opt. cemented (n = 386)	32 (8.3)	0.84	0.55 to 1.28	0.420	0.97	0.57 to 1.63	0.894
Optimal hybrid (n = 136)	14 (4.5)	1.07	0.59 to 1.93	0.832	0.74	0.29 to 1.93	0.542
Optimal cementless (n = 806)	69 (8.6)	0.87	0.63 to 1.21	0.410	0.82	0.53 to 1.27	0.381
Sub-opt cementless (n = 1060)	68 (6.4)	0.64	0.46 to 0.87	0.007	0.77	0.50 to 1.19	0.238
Optimal resurfacing (n = 96)	4 (4.2)	0.40	0.15 to 1.13	0.083	0.60	0.18 to 2.03	0.411
**Reoperation**							
Optimal cemented (n = 906)	21 (2.3)	1			1		
Sub-opt. cemented (n = 386)	6 (1.6)	0.67	0.27 to 1.66	0.383	0.85	0.31 to 2.34	0.749
Optimal hybrid (n = 136)	5 (3.7)	1.61	0.59 to 4.34	0.348	1.34	0.37 to 4.83	0.658
Sub-optimal hybrid (n = 424)	6 (1.4)	0.60	0.24 to 1.51	0.281	0.55	0.18 to 1.68	0.297
Optimal cementless (n = 806)	17 (2.1)	0.91	0.48 to 1.73	0.770	0.47	0.18 to 1.21	0.116
Sub-opt cementless (n = 1060)	18 (1.7)	0.73	0.39 to 1.37	0.328	0.72	0.33 to 1.56	0.409
Optimal resurfacing (n = 96)	1 (1.0)	0.44	0.06 to 3.33	0.430	1		-

OR–odds ratio, CI–confidence interval

Note: No predicted values are available for resurfacings in females (14 PROMs available only) and others resurfacing in males (2 PROMs only)

### Implant Revision Risk

When compared to the reference hip in females, the following had significantly higher revision risks: sub-optimal cemented (HR = 1.85, p<0.001), sub-optimal hybrid (HR = 1.68, p = 0.012), optimal cementless (HR = 2.22, p<0.001), sub-optimal cementless (HR = 3.60, p<0.001), and sub-optimal resurfacing (HR = 8.74, p<0.001). Optimal hybrid and optimal resurfacing had similar implant survival, but confidence intervals were wide for resurfacing (**Table 7**).

**Table 7:** Risk of revision following hip replacement in patients aged 60 years and over (simple and multivariable analyses).

	Simple			Multivariable		
	HR	95% CI	P value	HR	95% CI	P value
**Females (n = 47231)**						
Optimal cemented (n = 12788)	1			1		
Sub-optimal cemented (n = 9163)	1.77	1.28 to 2.44	0.001	1.85	1.31 to 2.61	<0.001
Optimal hybrid (n = 1238)	1.30	0.60 to 2.85	0.507	1.26	0.56 to 2.81	0.578
Sub-optimal hybrid (n = 6142)	1.73	1.19 to 2.52	0.004	1.68	1.12 to 2.52	0.012
Optimal cementless (n = 5303)	2.15	1.47 to 3.14	<0.001	2.22	1.48 to 3.34	<0.001
Sub-optimal cementless (n = 11559)	3.62	2.70 to 4.85	<0.001	3.60	2.63 to 4.94	<0.001
Optimal resurfacing (n = 166)	1.98	0.49 to 8.07	0.339	2.31	0.57 to 9.41	0.244
Sub-optimal resurfacing (n = 872)	7.66	5.21 to 11.3	<0.001	8.74	5.81 to 13.2	<0.001
**Males (n = 32544)**						
Optimal cemented (n = 7027)	1			1		
Sub-optimal cemented (n = 4510)	2.03	1.36 to 3.04	0.001	2.09	1.37 to 3.18	0.001
Optimal hybrid (n = 1150)	0.94	0.40 to 2.21	0.882	0.68	0.26 to 1.76	0.425
Sub-optimal hybrid (n = 3626)	1.47	0.92 to 2.37	0.108	1.28	0.78 to 2.11	0.327
Optimal cementless (n = 4564)	2.08	1.36 to 3.16	0.001	1.95	1.25 to 3.05	0.003
Sub-optimal cementless (n = 8167)	2.79	1.95 to 3.98	<0.001	2.53	1.74 to 3.68	<0.001
Optimal resurfacing (n = 3151)	3.30	2.23 to 4.88	<0.001	3.46	2.28 to 5.26	<0.001
Sub-optimal resurfacing (n = 349)	6.13	3.37 to 11.2	<0.001	6.21	3.36 to 11.5	<0.001

HR–hazard ratio, CI–confidence interval

For males, all implants except hybrids had significantly higher revision risk: sub-optimal cemented (HR = 2.09, p = 0.001), optimal cementless (HR = 1.95, p = 0.003), sub-optimal cementless (HR = 2.53, p<0.001), optimal resurfacing (HR = 3.46, p<0.001) and sub-optimal resurfacing (HR = 6.21, p<0.001) (**Table 7**).

### Material Costs

The reference (cemented) replacement in this analysis was the cheapest (most commonly paid total price £1138). Resurfacing implants ranged in total cost from £2018 to £2991. A cementless 36mm CoC implant cost the NHS between £2500 and £4285 (**Table 8**).

**Table 8:** Cost of specific hip implant combinations (NHS costs 2011/12).

Implant description		Stem		Femoral head		Cup		Ancillary items		Cost, mode / range (£)	Total cost* (£)
		Description	Cost (£)	Description	Cost (£)	Description	Cost (£)	Description	Cost (£)		
**CEMENTED Stryker Exeter V40 Contemporary**											
Most commonly used ‘optimal’ component set	Flanged cup / 28mm metal head	44/size 1	397.90 to 547.40	Stainless steel (Orthinox) V40 standard offset 28mm	145.00 to 257.60	Flanged cup	138.40 to 227.50	Heraeus Palacos R+Gentamycin antibiotic cement (4 mixes required)	26.75 /mix	**928.41** (898.88 to 1250.08)	**1138.09**
Alternative ‘optimal’	Flanged cup / 32mm ceramic head	44/size 1	397.90 to 547.40	Ceramic (Alumina) V40 standard offset 32mm	415.00 to 588.00	Flanged cup	138.40 to 227.50	DePuy Hardinge restrictor	22.00	**1343.41** (951.30 to 1580.48)	**1636.09**
Most commonly used ‘sub-optimal’	Hooded cup/26mm head	44/ size 1	397.90 to 547.40	Stainless steel (Orthinox) standard offset 26mm	138.40 to 227.50	Hooded cup	138.40 to 227.50	Biomet Optivac vacuum mixing and delivery system (2 required)	44.29 /kit	**928.41** (898.88 to 1250.08)	**1138.09**
**HYBRID Stryker Exeter V40 Trident**											
Most commonly used ‘optimal’ component set	Solid shell/ 36mm CoC	44/ size 1	397.90 to 547.40	Ceramic (Alumina) V40 standard offset 36mm	415.00 to 588.00	Ceramic 36mm liner plus PSL solid back shell	415.00 to 717.50 plus 432.40 to 646.10	Heraeus Palacos R+Gentamycin antibiotic cement (2 mixes required)DePuy Hardinge restrictorBiomet Optivac vacuum mixing and delivery system (1 required)	26.75 /mix22.0044.29	**1780.09** (1780.09 to 2618.79)	**2160.11**
Alternative ‘optimal’	Solid shell/32mm MoXLP	44/ size 1	397.90 to 547.40	Cobalt-chrome (Vitallium) V40 standard offset 32mm	145.00 to 271.60	X3 XLPE 32mm 10 degree liner plus PSL solid back shell	345.14 to 506.80 plus 432.40 to 646.10			**1465.00** (1440.23 to 2091.69)	**1782.00**
Most commonly used ‘sub-optimal’	Multi-hole shell/28mm MoP	44/ size 1	397.90 to 547.40	Cobalt-chrome (Vitallium) V40 standard offset 28mm	145.00 to 271.60	Conventional Polyethylene 28mm liner plus PSL 5-hole	230.09 to 375.20 plus 432.40 to 646.10	As above, plus 2 Stryker acetabular screws	40.00 to 51.10	**1405.18** (1405.18 to 2051.19)	**1710.22**
**RESURFACING Smith & Nephew Birmingham Hip Resurfacing**											
Optimal	Head size ≥48mm	-	-	BHR head	540.00 to 865.52	BHR cup	1050.00 to 1534.81	Stryker Antibiotic Simplex cement (1 mix required)	27.72	**1943.71** (1662.01 to 2472.34)	**2356.45**
Sub-optimal	Head size <48mm	-	-	BHR head	540.00 to 865.52	BHR cup	1050.00 to 1534.81				
**CEMENTLESS DePuy Corail Pinnacle**											
Most commonly used ‘optimal’ component set	28mm MoP	Size 11 KS	642.85 to 1118	Metal standard offset 28mm	130.53 to 227.00	Marathon 28mm PE neutral lip liner plus cluster-hole Duofix	252.43 to 439.00 plus 510.03 to 887.00	1 DePuy acetabular screw included	54.05	**1586.94** (1586.94 to 2722.10)	**1928.33**
Commonly used ‘sub-optimal’	36mm MoM	Size 11 KS	642.85 to 1118	Ultamet standard offset 36mm	249.55 to 434.00	Metal liner plus Sector cluster-hole Duofix	249.55 to 434.00 plus 510.03 to 887.00			**1790.78** (1703.08 to 2924.10)	**2172.94**
Commonly used ‘sub-optimal’	36mm CoC	Size 11 KS	642.85 to 1118	Ceramic 36mm standard offset	431.25 to 750.00	Ceramic liner plus Sector cluster-hole Duofix	428.38 to 745.00 plus 510.03 to 887.00			**2209.95** (2063.61 to 3551.10)	**2675.94**

CoC–ceramic-on-ceramic, MoXLP–metal-on-highly cross-linked polyethylene, MoP–metal-on polyethylene, MoM–metal-on-metal, PE–polyethylene.

Figures based on actual implant costs paid to manufacturers by NHS Wales (seven Trusts) and NHS Supply chain (30 Trusts in England). *Total cost is calculated using the mode cost plus NJR levy costs (£20) and Value Added Tax (20%). Note–very large Exeter stems (offset 44 sizes 4 and 5, and all 50 offset stems) increase cost by £614.27 (this represents less than 5% of all Exeter stems used) [32]

## Discussion

The reference implant (fully cemented, standard head size and conventional polyethylene cup) offered the lowest risk of implant failure at the lowest cost in patients over 60 years. No functional benefit of any implant was found in males relative to the reference implant; some differences for females were statistically significant but of unclear clinical importance. Readmission and reoperation rates were similar across all groups, suggesting there are no large variations in dislocation risk across implants. Notably higher costs and poorer implant survival was found when resurfacing and cementless implants were used. The findings of this summative evaluation of a range of hip replacements are contrary to current trends in surgery and may be useful for healthcare providers, surgeons and those commissioning hip replacement services.

As with all database analyses, the study design is observational and thus vulnerable to omitted variables. Implant choices in this cohort result from the interplay of patient, surgical and provider factors, and are not assigned randomly. Potentially important variables that were unavailable, such as radiological data, race, socioeconomic status, patient experiences, levels of perioperative pain and preoperative expectations, are known to influence outcome [41,42]; a large proportion of variation within the models in this study therefore remains unexplained.

The numbers within comparison groups were adequate in order to identify meaningful differences in PROMs, despite limiting to specific brands (to reduce the confounding effect of implant heterogeneity) [38]. Additionally, raw data from the NJR annual report suggests no other brands afford better implant survival than the commonest brands as used here [3]. Whilst the NJR only describes mid-term implant survival, there is currently no evidence to support the assertion that polyethylene-wear associated revision may occur in greater numbers beyond ten years, as other national registries established many decades ago show good long-term survival of cemented implants with polyethylene bearings (cemented polyethylene cup 90% survival at 16 years, compared with 85% for cementless, Swedish Annual Report 2011) [11]. A systematic review of world wide registry and cohort study data failed to show a benefit of other bearings when compared with MoP [6]. Furthermore, dislocation risk has been shown to be higher with CoC [43] and there are concerns surrounding metal wear debris reactions in patients with MoM implants, which has prompted a dramatic reduction in their use over the last five years [3,44].

This analysis covers an entire nation of surgeons and surgical units providing hip replacement, and therefore provides strong external validity. However, NJR data validity has been questioned; data loss and under-reporting of revision numbers remains a concern (although this should affect comparison groups equally). PROMs data are currently recorded only once post-operatively, at around six months following surgery, which may be too early to determine success of a joint replacement. Nevertheless, the greatest improvement in OHS occurs in the first three months, with no improvements seen beyond 12 months; results from this current study are therefore a reliable indication of longer-term outcome [45,46]. There may also be selection bias within the PROMs data; questionnaire response rates may vary across different ages, socioeconomic groups or race. The point at which a patient undergoes a hip procedure may also be different (reflecting the need to adjust for pre-operative scores), depending on age, expectations and occupation. Patients undergoing resurfacing tend to have higher pre-operative scores. This may in turn limit their ability to improve within the constraints of the current scoring systems, due to a ceiling effect of both the OHS and EQ5D index.

Pennington et al recently published a cost effectiveness paper using NJR, PROMs and implant cost data to compare types of hip replacement [47]. Hybrid implants were found to have the most cost-effective profile. Corroborating the findings presented in this current study, the authors found that cementless implants offered no benefit whilst being more costly. However, all brands within each hip replacement type were analysed collectively (using only MoP bearings), with no adjustment for the heterogeneity of implants. This limits the implications of their findings as pooling brands and configurations (when comparing procedures) may mask important differences between brand, configuration and procedure. However, Pulikottil-Jacob et al took this a step further by examining different types of hip replacement fixation and bearing, and found that available evidence does not support recommending a particular device on cost effectiveness grounds alone, although the authors did not examine PROMs or complication data [48].

Although hybrid implants have good implant survival in this current study, it must be stressed these results rely on rigid press-fit of the acetabular component into the bony socket without the need for supplementary screws to aid fixation. The use of multi-hole shells to allow supplementary screw fixation (as apposed to ‘solid’ shells, without holes) have a 37% higher risk of revision [34]. Whilst a cemented procedure will have reproducible results, adequate cementless cup fixation may be more difficult to achieve.

The fully cementless implant analysed here has a 1.9 to 3.6 times higher revision risk than the standard cemented implant. Although there was a higher OHS improvement (1.6 points) in females, this is below the clinical important threshold of 3 to 5 points suggested by the OHS designers [39,49]. Proponents of fully cementless procedures argue that the costs may actually be lower than those of cemented implants, as cementation requires greater operative time [50]. Although we chose to analyse the commonest cementless implant, we acknowledge that others may have lower costs. We have assumed that implant specific costs approximate to the incremental costs of different implants. There remains no good evidence of improved theatre efficiency for cementless implants in the literature; savings of 15 to 20 minute per case have been suggested [50,51,52], but equating this to monetary savings is only credible when extra replacements are actually performed within an operating schedule. Additionally, our analysis is likely to understate the true incremental costs of implants: subsequent revision surgery (which occurs more commonly with cementless and resurfacing procedures) would increase the overall costs of these types relative to cemented implants. One study found that annual hip replacement costs in the US (where cementless implants are used almost exclusively) could be reduced by $2billion if there was a joint registry comparable to the Swedish registry (enabling reductions in revision rates) [53]. The use of cement on the femoral side has many advantages that outweigh the disadvantage of a slightly longer operative time [28], and the available literature suggests that cemented fixation of acetabular components is more reliable than cementless beyond the first postoperative decade [14].

This study demonstrates no benefit of a resurfacing procedure in patients over 60 years across any of the domains studied in this analysis. Given the high failure rates, the risks of local and systemic complications, and the long-term concerns surrounding these implants, including a medical device warning and mandatory annual follow-up, there appears to be no routine place for a resurfacing procedure in patients over 60 years [44,54]. Even in the ideal resurfacing patient (a young male), Heintzbergen et al showed that absolute differences in cost-utility were small when a BHR was compared to conventional hip replacement [55]. A dramatic fall in the use of resurfacings, with use predominantly in young males during 2011 suggests surgeons practising in England and Wales are responding to the evidence [3].

Long-term observational studies of mortality after hip replacement suggest a higher risk of death when cement is used, but these fail to account for the confounding effect of true patient differences and provide no logical reason for the increased death rate many years after cementation [56,57]. However, an analysis of over 400,000 hip replacements performed in England and Wales between 2003 and 2011, using a combination of NJR and hospital episodes data (allowing for extensive patient and provider variable adjustment) found the use of hip replacement type to have no impact on mortality at 90 days following surgery [58], implying that cement pressurisation at the time of surgery does not influence surgery-associated mortality.

In the past decade hip surgeons have been guilty of using implants with limited long-term evidence at great expense to the NHS and other healthcare providers (as a result of costs incurred initially and at revision surgery), and with significant adverse impact on patient outcomes [59]. Fordham et al stated that the most cost-effective implants are those with the best survival rates (and hence the fewest revisions), with the best patient outcomes and the least cost [1]. Within this multi-outcome study of national data, a cemented stem with a cemented polyethylene cup and a standard sized head offered similar outcomes to other implants, but with lower revision risk and at the lowest costs. This category of implant should be the gold standard for hip replacement, and used for comparisons with new implants within future robust, randomised clinical trials. Uptake of new implants should depend upon evidence of reduced revisions, patient morbidity and healthcare resource use.

The proliferation of hip replacement options has meant that any analysis aiming to determine ‘optimal’ hip replacement is inherently complex. However, the intention of this study was to provide a summative evaluation of a range of hip replacements for the patient over 60 years with hip OA. This type of evaluation is crucial to inform commissioning decisions by helping to answer the question 'what is the most cost-effective hip replacement?’ We believe the findings of this paper will appeal to commissioners, surgeons, healthcare management and the broader medical community striving to delivery high quality and cost effective healthcare.

## Supporting Information

S1 Table Summary of the demographic and surgical variables available for analysis. (PDF)

S2 Table Variables included in the competing risks survival model. (PDF)

S3 Table Competing risks survival modelling of hip type using different variable sets. (PDF)

S4 Table Variables included in the change score analysis of covariance models. (PDF)

S5 Table Variables included in the complications multivariable logistic regression models. (PDF)

S1 Text Supplementary methodology. (PDF)
