# Supplementary material for: Implant Optimisation for Primary Hip Replacement in Patients over 60 Years with Osteoarthritis: A Cohort Study of Clinical Outcomes and Implant Costs Using Data from England and Wales
Source: PLoS One. 2015 Nov 12;10(11):e0140309. doi: 10.1371/journal.pone.0140309 (PMC4643061; doi:10.1371/journal.pone.0140309)
Supplement: S1 Table — (PDF) [file pone.0140309.s001.pdf]

S1 Table. Summary of the demographic and surgical variables available for analysis

	Source	Description
**Patient factors**		
Age (years)	NJR/PROMs	
Sex	NJR/PROMs	
American Society of Anaesthesiology (ASA) grade	NJR	Grades 1 to 4
Body mass index (BMI) (kg/m^2)	NJR	Only BMI within 15 kg/m^2 to 60 kg/m^2 included
Comorbidities	PROMs	Recorded by patients as part of the pre-operative PROMs questionnaire. Nine co-morbidities: i) ischaemic heart disease, ii) respiratory disease, iii) diabetes, iv) hypertension, v) kidney disease, vi) liver disease, vii) circulatory problems, viii) cancer, ix) depression
Pre-operative general health	PROMs	Indicates the patient's perception of their own general health with five options: i) excellent, ii) very good, iii) good, iv) fair, v) poor
Pre-operative disability	PROMs	Indicates whether the patient considers themselves to have a disability
Pre-operative Oxford Hip Score (OHS)	PROMs	Derived from adding the points (0 to 4) together from the response to hip symptom-specific questions on a scale of 0 to 48 (0 worst, 48 best)
Pre-operative EQ5D Visual Analogue Score	PROMs	Indicates how well the patient feels on the day of completing the questionnaire on a scale of 0-100 (0 worst, 100 best)
Pre-operative EQ5D index	PROMs	Single summary score derived from EQ5D profile (based on response to 5 questions) by applying a formula with appropriate operation specific weightings
**Surgical factors**		
Lead surgeon grade	NJR	Consultant or other
Surgeon volume	NJR	i) Low, ii) medium, iii) high
Approach	NJR	i) Posterior, ii) direct lateral, ii) other
Patient position	NJR	i) Lateral, ii) supine, iii) not recorded
Type of replacement	NJR	i) Best cemented, ii) Other cemented, iii) Best hybrid, iv) Other hybrid, v) Best cementless, vi) Other cementless, vii) Best resurfacing, viii) Other resurfacing
Anaesthesia	NJR	i) Regional only, ii) general only, iii) general and regional
Chemical venous thromboembolism prophylaxis	NJR	Intended prophylaxis as recorded at time of operation: i) aspirin only, ii) LMWH only, iii) other, iv) none, v) not recorded
Mechanical venous thromboembolism prophylaxis	NJR	Intended prophylaxis as recorded at time of operation: i) Compression stockings (CS) only, ii) combination CS/mechanical pump, iii) foot pump only, iv) intermittent calf pump only, v) other, vi) none, vii) not recorded
Time from operation to post-operative PROMs completion	PROMs	Calculated from the date of operation as recorded on the NJR database to the date of post-operative PROMs as recorded on the questionnaire
