# Supplementary material for: Implant Optimisation for Primary Hip Replacement in Patients over 60 Years with Osteoarthritis: A Cohort Study of Clinical Outcomes and Implant Costs Using Data from England and Wales
Source: PLoS One. 2015 Nov 12;10(11):e0140309. doi: 10.1371/journal.pone.0140309 (PMC4643061; doi:10.1371/journal.pone.0140309)
Supplement: S2 Table — (PDF) [file pone.0140309.s002.pdf]

S2 Table. Variables included in the competing risks survival model

	**Females**		**Males**	
	Simple	Multivariable	Simple	Multivariable
Age	<0.001	-	0.001	-
ASA grade	0.371	0.046	0.868	-
BMI	0.003	(0.003)*	0.003	(0.036)*
Hip type	<0.001	<0.001	<0.001	<0.001
Approach	0.634	-	0.961	-
Surgeon grade	0.496	-	0.068	-
Surgeon volume	0.513	-	0.675	
Anaesthesia type	0.790	-	0.088	0.095
Mechanical VTE prophylaxis	0.376	-	0.019	-
Chemical VTE prophylaxis	0.044	0.024	0.012	0.007

BMI – body mass index

* - BMI data available for only 20708 (44%) females and 14048 (43%) therefore excluded from the model. Results in appendix table 3 show similar results for models with and without BMI.
