# Supplementary material for: Implant Optimisation for Primary Hip Replacement in Patients over 60 Years with Osteoarthritis: A Cohort Study of Clinical Outcomes and Implant Costs Using Data from England and Wales
Source: PLoS One. 2015 Nov 12;10(11):e0140309. doi: 10.1371/journal.pone.0140309 (PMC4643061; doi:10.1371/journal.pone.0140309)
Supplement: S3 Table — (PDF) [file pone.0140309.s003.pdf]

S3 Table. Competing risks survival modelling of hip type using different variable sets

	Simple (HR, 95% CI)	BMI included (HR, 95% CI)	BMI excluded (HR, 95% CI)
**Females**			
Optimal cemented	1	1	1
Sub-opt. cemented	1.77 (1.28 to 2.44, p=0.001)	2.48 (1.40 to 4.39, p=0.002)	1.85 (1.31 to 2.61, p<0.001)
Optimal hybrid	1.30 (0.60 to 2.85, p=0.507)	0.85 (0.19 to 3.74, p=0.831)	1.26 (0.56 to 2.81, p=0.578)
Sub-optimal hybrid	1.73 (1.19 to 2.52, p=0.004)	1.58 (0.81 to 3.06, p=0.180)	1.68 (1.12 to 2.52, p=0.012)
Optimal cementless	2.15 (1.47 to 3.14, p<0.001)	1.68 (0.85 to 3.30, p=0.134)	2.22 (1.48 to 3.34, p<0.001)
Sub-opt cementless	3.62 (2.70 to 4.85, p<0.001)	3.56 (2.16 to 5.86, p<0.001)	3.60 (2.63 to 4.94, p<0.001)
Optimal resurfacing	1.98 (0.49 to 8.07, p=0.339)	3.77 (0.55 to 25.9, p=0.177)	2.31 (0.57 to 9.41, p=0.244)
Sub-opt resurfacing	7.66 (5.21 to 11.3, p<0.001)	9.21 (4.57 to 18.6, p<0.001)	8.74 (5.81 to 13.2, p<0.001)
**Males**			
Optimal cemented	1	1	1
Sub-opt cemented	2.03 (1.36 to 3.04, p=0.001)	1.24 (0.53 to 2.89, p=0.615)	2.09 (1.37 to 3.18, p=0.001)
Optimal hybrid	0.94 (0.40 to 2.21, p=0.882)	0.78 (0.22 to 2.80, p=0.704)	0.68 (0.26 to 1.76, p=0.425)
Sub-optimal hybrid	1.47 (0.92 to 2.37, p=0.108)	1.02 (0.43 to 2.42, p=0.971)	1.28 (0.78 to 2.11, p=0.327)
Optimal cementless	2.08 (1.36 to 3.16, p=0.001)	1.77 (0.90 to 3.50, p=0.100)	1.95 (1.25 to 3.05, p=0.003)
Sub-opt cementless	2.79 (1.95 to 3.98, p<0.001)	2.29 (1.19 to 4.42, p=0.013)	2.53 (1.74 to 3.68, p<0.001)
Optimal resurfacing	3.30 (2.23 to 4.88, p<0.001)	2.81 (1.29 to 6.18, p=0.010)	3.46 (2.28 to 5.26, p<0.001)
Sub-opt resurfacing	6.13 (3.37 to 11.2, p<0.001)	7.36 (2.54 to 21.3, p<0.001)	6.21 (3.36 to 11.5, p<0.001)

HR – hazard ratio, CI – confidence intervals, BMI – body mass index

* BMI data available for 20708 of 47231 procedures in females (44%) and 14048 of 32544 procedures in males (43%)
