# Supplementary material for: Implant Optimisation for Primary Hip Replacement in Patients over 60 Years with Osteoarthritis: A Cohort Study of Clinical Outcomes and Implant Costs Using Data from England and Wales
Source: PLoS One. 2015 Nov 12;10(11):e0140309. doi: 10.1371/journal.pone.0140309 (PMC4643061; doi:10.1371/journal.pone.0140309)
Supplement: S4 Table — (PDF) [file pone.0140309.s004.pdf]

S4 Table. Variables included in the change score analysis of covariance models

	**Oxford hip score change**		**EQ5D index change**	
	BMI included	BMI excluded	BMI included	BMI excluded
**Females**				
Hip type	0.003	0.001	0.519	0.119
Age	<0.001	<0.001	0.039	0.036
BMI	<0.001	-	<0.001	-
Preoperative OHS	<0.001	<0.001	-	-
Preoperative EQ5D index	<0.001	<0.001	<0.001	<0.001
Preoperative general health	<0.001	<0.001	<0.001	<0.001
Preoperative disability	<0.001	<0.001	<0.001	0.001
Circulatory problems	<0.001	<0.001	<0.001	<0.001
History of depression	0.007	<0.001	<0.001	<0.001
History of heart disease	0.066	-	-	-
History of stroke	0.001	0.008	0.011	-
History of cancer	-	-	0.047	-
Approach	<0.001	<0.001	<0.001	<0.001
Surgeon grade	0.017	-	0.002	0.018
Time from op to PROMs completion	0.074	0.003	0.004	0.006
Goodness of fit of model, adjusted R^2 (number in model)	40% (3091)	37% (4804)	63% (2937)	60% (4585)
**Males**				
Hip type	0.901	0.270	0.372	0.651
Age	0.030	-	-	-
BMI	<0.001	-	0.061	-
Preoperative OHS	<0.001	<0.001	0.001	<0.001
Preoperative EQ5D index	-	-	<0.001	<0.001
Preoperative health scale	0.054	0.034	0.007	<0.001
Preoperative general health	<0.001	<0.001	<0.001	<0.001
Preoperative disability	<0.001	<0.001	<0.001	<0.001
Circulatory problems	<0.001	<0.001	<0.001	<0.001
History of depression	0.002	0.003	<0.001	<0.001
History of lung disease	0.050	-	-	-
History of stroke	0.016	0.035	0.047	0.041
Approach	0.002	0.002	0.002	0.036
Anaesthesia	0.037	0.045	0.034	0.078
ASA group	-	-	0.008	<0.001
Goodness of fit of model, adjusted R^2 (number in model)	42% (2130)	42% (3410)	61% (1999)	60% (3179)

BMI – body mass index.
