# Supplementary material for: Implant Optimisation for Primary Hip Replacement in Patients over 60 Years with Osteoarthritis: A Cohort Study of Clinical Outcomes and Implant Costs Using Data from England and Wales
Source: PLoS One. 2015 Nov 12;10(11):e0140309. doi: 10.1371/journal.pone.0140309 (PMC4643061; doi:10.1371/journal.pone.0140309)
Supplement: S5 Table — (PDF) [file pone.0140309.s005.pdf]

S5 Table. Variables included in the complications multivariable logistic regression models

	**Readmission**		**Reoperation**	
	Females	Males	Females	Males
Hip type	0.638	0.230	0.122	0.089
Age	0.012	0.036	-	0.058
BMI	0.045	0.001	-	0.044
ASA group	-	-	0.017	-
Preoperative Oxford hip score	0.001	-	-	-
Preoperative EQ5D index	-	0.065	0.001	-
Preoperative VAS	-	-	-	-
Preoperative general health	-	-	-	-
Preoperative disability	-	-	-	-
History of kidney disease	-	-	-	0.029
Diabetes	-	-	0.026	-
Circulatory problems	-	-	-	-
History of heart disease	-	-	0.052	0.069
History of depression	0.013	-	-	-
History of liver disease	-	0.069	-	-
History of stroke	0.034	-	-	-
Approach	-	0.015	-	-
Surgeon grade	0.022	0.039	-	-
Chemical VTE prophylaxis	-	-	-	=
Time from operation to PROMs completion	0.009	-	-	-
Number in model	3421	2314	5035	2393

BMI – body mass index
