# Supplementary material for: Implant Optimisation for Primary Hip Replacement in Patients over 60 Years with Osteoarthritis: A Cohort Study of Clinical Outcomes and Implant Costs Using Data from England and Wales
Source: PLoS One. 2015 Nov 12;10(11):e0140309. doi: 10.1371/journal.pone.0140309 (PMC4643061; doi:10.1371/journal.pone.0140309)
Supplement: S1 Text — (PDF) [file pone.0140309.s006.pdf]

Supplementary material

To carry out linkage between NJR and PROMs databases a number of criteria were used: firstly, to ensure correct matching, two unique identifiers (NJR and procedure numbers) recorded in both datasets were used; secondly, the operation date recorded by the patient in the PROMs data had to be within +/-30 days of the operation date recorded on the NJR record, to ensure the patient was scoring the same procedure. Procedures with PROMs data that were missing, undated, dated more than 12 months prior to or following the operation, or non-identical duplicates were excluded; for identical duplicates the first record was retained for analysis. Where the presence of a comorbidity was sought in the questionnaire but left blank by the patient, it was assumed to be absent.

The reliability of the multivariable statistical models was explored in a number of ways: covariates found not to be statistically significant were excluded from the model, based on statistical entry ($p<0.05$) criteria; the same covariates were fitted forward and reverse stepwise manually to ensure findings were not qualitatively affected in the final model, with any inconsistency reported. The final models were then re-evaluated as a directly entered model (non-stepwise), assessed by exploring 2-way interactions between covariates and, for the survival analysis, assessed for the constant proportionality over time assumption.

Tests for interaction (multiplicative) between covariates and for time-dependency were not statistically significant. Forward and reverse stepwise model construction

and varying significance thresholds led to the same final models. Variables included in the competing risks model are shown in **S2 table**. BMI was selected as a variable within the competing risks survival model for males. However this approach excluded 63% of data. BMI was therefore excluded and the model was constructed with age and ASA group. The output from these models (simple and multivariable with either BMI or age and ASA group included) is shown in **S3 table**. Variables included in the statistical models, and their significance levels within the final models, are shown in **S4 and S5 tables**.
